# Glyphosate Sensor Based on Nanostructured Water-Gated CuO Field-Effect Transistor

**DOI:** 10.3390/s22228744

**Published:** 2022-11-12

**Authors:** Andrejs Ogurcovs, Kevon Kadiwala, Eriks Sledevskis, Marina Krasovska, Valdis Mizers

**Affiliations:** 1 Institute of Solid State Physics, University of Latvia, Kengaraga Street 8, LV-1063 Riga, Latvia; 2 G. Liberts’ Innovative Microscopy Centre, Department of Technology, Institute of Life Sciences and Technology, Daugavpils University, Parades Street 1A, LV-5401 Daugavpils, Latvia

**Keywords:** copper oxide, thin-film transistor, glyphosate, nanostructures, water-gated field effect transistor

## Abstract

This research presents a comparative analysis of water-gated thin film transistors based on a copper oxide (CuO) semiconductor in the form of a smooth film and a nanostructured surface. A smooth CuO film was deposited through reactive magnetron sputtering followed by annealing in atmosphere at a temperature of 280 ${}^{\circ }$C. Copper oxide nanostructures were obtained by hydrothermal synthesis on a preliminary magnetron sputtered 2 nm thick CuO precursor followed by annealing at 280 ${}^{\circ }$C. An X-ray diffraction (XRD) analysis of the samples revealed the presence of a tenorite (CuO) phase with a predominant orientation of (002). Scanning electron microscopy (SEM) and atomic force microscopy (AFM) studies of the samples revealed a highly developed surface with crystallites having a monoclinic syngony and dimensions of 15–20 nm in thickness, 150 nm in length, and 100 nm in height relative to a 2.5 nm height for the CuO crystallites of the smooth film. Electric measurements of the studied devices revealed typical current–voltage characteristics of semiconductors with predominant hole conductivity. The maximum ON/OFF ratio at a rain-source voltage of 0.4 volts and −1.2 volts on the gate for a smooth film was $10^{2}$, and for a nanostructured transistor, it was $10^{3}$. However, a much stronger saturation of the channel was observed for the nanostructured channel than for the smooth film. A test solution containing glyphosate dissolved in deionized water in three different concentrations of 5, 10, and 15 μmol/L was used during the experiments. The principle of operation was based on the preliminary saturation of the solution with Cu ions, followed by the formation of a metal–organic complex alongside glyphate. The glyphosate contents in the analyte led to a decrease in the conductivity of the transistor on the axis of the smooth film. In turn, the opposite effect was observed on the nanostructured surface, i.e., an increase in conductivity was noted upon the introduction of an analyte. Despite this, the overall sensitivity of the nanostructured device was twice as high as that of the device with a thin film channel. The relative changes in the field-effect transistor (FET) conductivity at maximum glyphosate concentrations of 15 μmol/L reached 19.42% for the nanostructured CuO film and 3.3% for the smooth film.

## 1. Introduction

Glyphosate (N-(phosphonomethyl)glycine), widely known as Roundup™, is the most commonly used herbicide in many countries [1,2,3,4]. Recent research has proven that even in low dosages, glyphosate negatively affects a range of insects, earthworms, and microorganisms. The contamination of soil and groundwater by glyphosate leads to environmental imbalances: a decrease in the number of certain types of bacteria causes the uncontrolled reproduction of other, often pathogenic, microorganisms [5]. In addition, glyphosate negatively affects the health of plants, making them susceptible to various fungal diseases, as well as suppressing plant growth and causing genetic changes within them [6]. Following the treatment of fields with glyphosate, the latter is absorbed by the cultivated plants, also becoming incorporated into the soil and sewage in significant amounts and spreading over considerable distances. In the process of degradation of glyphosate, a shorter aminomethylphosphonic acid (AMPA) molecule is formed, which also possesses herbicidal effects and is more toxic than pure glyphosate. In Europe, the permissible level of pollutants does not exceed 0.1 μg/L [7]. In the United States, a fairly high maximum glyphosate level (700 μg/L) is allowed in drinking water. However, the highest allowed concentration of glyphosate in drinking water is found in Australia (1 mg/L) [8]. Until now, it has been argued that the toxicity of glyphosate to humans is very low because humans and other mammals lack the 5-enolpyruvylshikimate-3-phosphate-EPSP synthase enzyme, which is the main target of glyphosate in plants. However, recent studies do not support this view. Recent research has mentioned the cumulative effect and long-term intoxication risks associated with glyphosate [9]. Even in levels lower than needed for herbicidal effects, glyphosate and its metabolites can cause carcinogenic [10] and genotoxic effects [11], as well as leading to endocrine disorders [12,13] and infertility [14]. Comparative studies of glyphosate and its commercial product (RoundUp^TM^) in in vitro experiments on human cells have shown that the toxicity of glyphosate is 2 g/L, and the toxicity of RoundUp400 and 450 is 0.001 g/L [15]. The higher toxicity of the final product is explained by the fact that it contains additional adjuvant substances aimed at accelerating the absorption of glyphosate by plants and enhancing its herbicidal action. Residual traces of glyphosate and its metabolites can be found in food and drink, soil, water, and dust, so everyone can potentially be exposed to its toxic effects [16]. Therefore, the development and implementation of a simple sensor for detecting glyphosate at relatively low concentrations is an important task. Different types of sensors for glyphosate detection have been described in recent publications [17,18]. The most common detection methods are chromatographic [19], colorimetric [20], and amperometric [21]. Electrochemical sensors have previously proven to be effective tools for the determination of glyphosate [22]. The functionalization of the surface of the working electrodes with enzymes (in particular horseradish peroxidase) has previously been applied to ensure selectivity [23]. Enzyme sensors have a number of disadvantages; for example, sensors are expensive to manufacture, and the enzymes are thermally and chemically unstable due to their nature and can easily be damaged during storage, transportation and exploitation. The disadvantages of enzymatic sensors can be avoided by using non-enzymatic sensors that act through direct enzyme-like catalytic reactions between the sensor material and the analyte. The reaction of glyphosate with copper oxide (CuO) is widely used to detect the former [24].

Popular methods for obtaining CuO nanostructures include the thermal oxidation of Cu in an oxygen atmosphere, electrochemical and sonochemical deposition, and the sol-gel method. However, in recent years, the method involving the hydrothermal synthesis of nanostructures has been most widely used. It does not require expensive reagents, complex equipment, or extreme conditions (such as a vacuum and high temperatures) and is, moreover, environmentally friendly. Due to the low synthesis temperatures, materials of practically any composition, including flexible polymers, can be used as substrates. In addition, an indisputable advantage is that the parameters of the obtained nanostructures and their morphology can be controlled simply by changing the synthesis parameters (temperature, time, composition of the working solution, seed layer, etc.). CuO is a promising material for use as a non-enzymatic sensor material. It has remarkable catalytic and electronic properties. It is known that surface conductivity plays an important role in any sensor system based on electrochemical detection. It can be seen from publications [25,26] that CuO nanomaterials are represented in various morphologies. All of them have a very developed surface and, as a result, improved electrochemical characteristics and can serve as a suitable alternative to traditional electrode materials. In addition to the above advantages, it should be noted that, unlike most metal oxides, CuO can be obtained by one-step hydrothermal synthesis at relatively low temperatures (less than 100 degrees Celsius) without the need for calcining the samples. This way of nanostructure synthesis makes it possible to use a substrate of various materials sensitive to high temperatures, including flexible polymers.

A relatively new type of sensor consists of sensors based on field-effect transistors (FET), in particular electrolyte-gated field effect transistors. This type of sensor has a number of significant advantages, and therefore, it is currently being widely developed. In such devices, the semiconductor comes into direct contact with the analyte without requiring the prior isolation of the transistor semiconductor channel, leading to the formation of an electrical double layer (EDL) at the gate/electrolyte/semiconductor interfaces [27]. Since the EDL acts as an efficient gate insulator, a significant reduction in operating voltages can be observed. A gate electrode is commonly used to modulate the electronic response of the channel. To gain a higher selectivity and sensitivity, a process of functionalization with amino acids, antibodies, and enzymes is applied to the surface of the channel by adsorption or linker molecules. When the target molecule binds to a bioreceptor on the sensor surface through covalent bonding, electrostatic, or Van der Waals forces, a change in the net electric charge in the semiconductor channel can occur [28]. Field-effect transistors, as well as electrochemical sensors, can be non-enzymatic. In these cases, the surface is modified through the use of nanostructures, and sensitivity is enhanced due to the presence of chemical reactions between the analyte molecules and the sensor substance. Thin-film sensors are widely used; however, the use of nanostructured coatings can significantly increase the sensitivity of the sensor [29]. Nanostructures, due to their developed surfaces, significantly enhance the effective surface of the sensor due to the formation of a larger number of adsorption bonds [29]. We have proved that the use of CuO nanostructures in the detection of H2O2 increases the sensitivity of the sensor several times compared to smooth films. In addition, this study shows an important effect of nanostructure morphology on sensor sensitivity and confirms that increasing porosity (resulting in an increase in working surface area) significantly increases sensor sensitivity [30]. An even more significant increase in sensitivity can be obtained using the assisted growth template, as well as additional functionalization of nanostructures, for example, using PAM [24]. With its high sensing property, the materials in this work are promising for future super-flexible and wearable devices for farm workers. Therefore, the possible routes can be provided for future application, as revealed in the following papers [31,32].

The aim of this study was to develop an enzyme-free FET sensor to determine the presence of glyphosate in water samples. As part of the study, petal-like nanostructures of CuO were obtained by hydrothermal synthesis and were subsequently used as a working coating of the thin-film transistor (TFT) channel. The resulting non-enzymatic sensor was shown to be highly sensitive to glyphosate. An increased sensitivity of the nanostructured CuO coating was also shown relative to that of the thin-film coating.

## 2. Materials and Methods

### 2.1. Reagents

During the experiments, the following reagents were used: isopropanol (Chempur, Twickenham, England, Purity: 99.7%, CAS No.: 67-63-0), stainless steel masks (Shandong Detong Stainless Steel Co., Ltd., Jinan, PRC, Grade: AISI 304), chromium tartget (Kurt J. Lesker Company, Jefferson Hills, PA, USA, Purity: 99.95%, CAS No.: 7440-47-3), gold target (Ted Pella Inc. Redding CA, USA, Purity: 99.99%, CAS No.: 7440-57-5), tantalum target (Kurt J. Lesker Company, Jefferson Hills, PA, USA, Purity: 99.95%, CAS No.: 7440-25-7), copper target (Kurt J. Lesker Company, Jefferson Hills, USA, Purity: 99.999%, CAS No.: 7440-50-8), copper nitrate (Cu(NO${}_{3}$)${}_{2}$) (Lach-Ner, Ltd., Neratovice, Czech Republic, Purity: 99.9%, CAS number: 13778-31-9), C${}_{6}$H${}_{12}$N${}_{4}$ (HMT) (Lach-Ner, Ltd., Neratovice, Czech Republic, Purity: 99.5%, CAS No.: 100-97-0), NH${}_{3}$ (Chempur, Twickenham, England, Purity: 23.0%, CAS No.: 1336-21-6), glyphosate (Sigma-Aldrich, Saint Louis, MO, USA, PESTANAL^®^, analytical standard, CAS No.: 1071-83-6), DI water (Sigma-Aldrich, Saint Louis, USA, CAS No.:7732-18-5).

### 2.2. Sample Preparation

Polyimide substrates (15 × 15 mm in length-width and 0.2 mm in thickness) were fabricated by laser cutting and subsequently washed in isopropanol in an ultrasound bath for 15 min. The sputtering masks consisting of 0.2 mm-thick stainless steel were produced using a laser demetallization method. All parts of the TFTs were fabricated entirely by magnetron sputtering. The electrodes of the device consisted of a 3 nm thick chromium layer, providing better adhesion, and a 50 nm thick gold layer. A 60 nm thick Ta${}_{2}$O${}_{5}$ insulator layer was deposited by reactive magnetron sputtering from tantalum in an oxygen atmosphere to protect specific parts of the device from contact with the analyte during the operation. The CuO semiconductor layer was deposited onto the electrodes by reactive magnetron sputtering from a Cu metal target in an oxygen atmosphere. A 40 nm thick CuO film was used for the FET device with a smooth surface. For the preparation of series of the FET device with nanostructured channels, a 2 nm thick CuO layer was deposited as a precursor layer for the subsequent selective hydrothermal synthesis of CuO nanostructures. Hydrothermal synthesis was carried out in an aqueous solution containing 5 mM of Cu(NO${}_{3}$)${}_{2}$ (CAS number: 13778-31-9), 5 mM of C${}_{6}$H${}_{12}$N${}_{4}$ (HMT), and 2.5 mM of NH${}_{3}$ (25%). The synthesis process took 40 min at 80 ${}^{\circ }$C and atmospheric pressure. Next, all obtained sets of samples were annealed at 280 ${}^{\circ }$C for 90 min to improve the crystallinity and stoichiometry of the CuO and Ta${}_{2}$O${}_{5}$ layers. In total, 45 thin-film transistors with a channel in the form of a smooth CuO film and 45 devices with a nanostructured channel were fabricated.

### 2.3. Materials Characterization

The morphological aspects of the films were assessed using a TESCAN MAIA 3 TRIGLAV (Tescan, Brno, The Czech Republic) scanning electron microscope (SEM) and a PARK NX10(Park Systems, Suwon, Korea) atomic force microscope (AFM) in non-contact mode. X-ray diffraction (XRD) spectra of the CuO films were obtained with a Rigaku Miniflex 600 X-ray diffractometer (Rigaku Corporation, Tokyo, Japan). The electrochemical measurements were carried out with a Zahner Zennium (Zahner-Elektrik GmbH&Co., Kronach–Gundelsdorf, Germany) electrochemical station. Cyclic voltammetry with a voltage range of ±2.5 V and a scanning speed of 50 mV/s were used to determine the electrochemical window of Au/Cu in deionized water. The electric properties of the obtained TFTs were investigated using two Keithley 2400 (Keithley Instruments, LLC, Solon, OH, USA) source-meter units.

### 2.4. Experiments with Glyphosate

**Figure 1:**
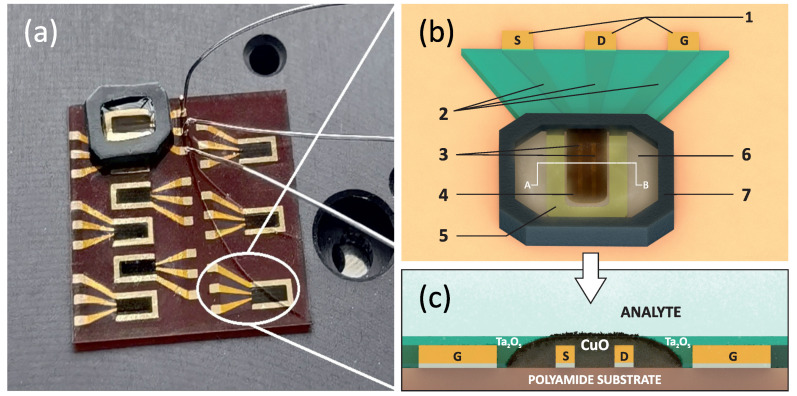
(**a**) An actual image of the polyimide substrate with CuO FET array and attached polysiloxane cuvette. (**b**) A schematic view of a single FET element: 1—gate, source and drain contact pads; 2—gate, source and drain electrodes passivated with Ta${}_{2}$O${}_{5}$; 3—CuO layer on top of the drain-source channel; 4—CuO layer; 5—the gate electrode; 6—analyte (glyphosate aqueous solution); 7—polysiloxane cuvette. (**c**) Cross section view of the investigated water-gated CuO FET-based glyphosate sensor along line A–B.

To maintain a constant analyte volume and coverage area, a special silicon container was fabricated, which was capable of containing up to 10 μL of analyte on top of the TFT (Figure 1a). The interaction of glyphosates of various concentrations with the nanostructures was studied in real-time mode. Five microliters of water were placed in the aforementioned cuvette, and potentials of 0.2 and −0.5 V were applied to the source-drain electrodes and at the gate electrode for both types of TFT devices. After 90 s, 5 μL of the analyte was added. The total duration of the experiment was nine minutes for each sample. Glyphosate aqueous solutions were used as an analyte at three concentrations: 5, 10, and 15 μmol/L. All measurements were carried out at an ambient temperature of 21 ${}^{\circ }$C and a humidity of 33%.

## 3. Results and Discussion

### 3.1. Device Geometry

Figure 1a shows a photograph of an actual substrate with an array of nine water-gated transistors on the probe station with attached Pt/Ir alloy leads and a polysiloxane cuvette capable of holding up to 10 μL of analyte. A schematic of a single device unit, including all the components, is shown in Figure 1b. The length of the transistor channel is 110 μm and the width is 2100 μm, thus having a channel width/length ratio equal to 19.09. The gate electrode area is 1.83 mm^2^, the area of the CuO layer is 1.5 mm^2^, and the distance between the gate and semiconductor layer is 150 μm. The planar gate transistor configuration provides a fixed gate-to-electrolyte contact area and distance to the transistor channel, thereby minimizing electrical variation and increasing the reproducibility of the experiment [33]. Transistors of this type are not sensitive to changes in the volume of the analyte, which allows more precise measurements in real-time mode compared to the systems with an external immersible gate electrode. In such type of systems, the degree of immersion of the gate electrode changes when an additional portion of the analyte is added, which affects the conductivity of the transistor channel due to variations in the capacitance of the electric double layer (EDL) [34], thereby complicating the interpretation of the results. In total, 45 TFTs with a smooth film and 45 nanostructured TFTs were fabricated. The dispersion of electrical characteristics for smooth CuO film devices was within 0.4% from device to device and 2.3% for nanostructured samples (initial resistivity tests TFT channel without an electrolyte (water)).

### 3.2. CuO Surface Structure

**Figure 2:**
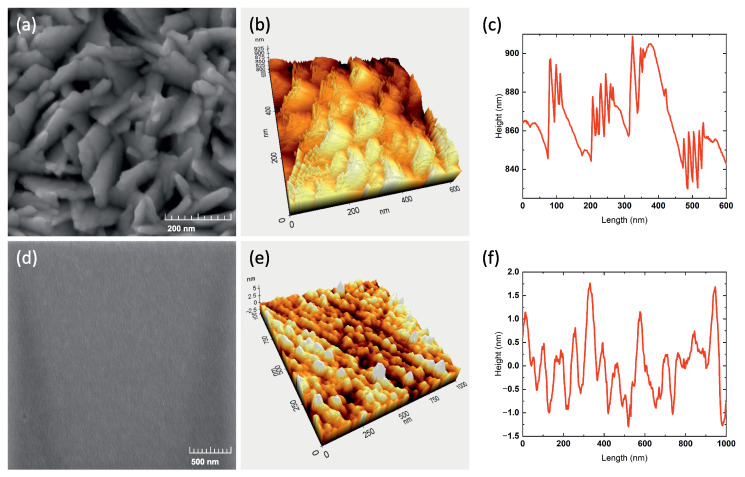
The figure represents SEM and AFM measurement results for nanostructured surface (**a**–**c**) and smooth film (**d**–**f**).

The SEM and AFM measurement results presented in Figure 2d–f reveal the structural aspects of the magnetron-sputtered CuO film. The surface of the film is smooth, consisting of grains with an average size of 80 nm in diameter and 2.5 nm in height.

The SEM analysis of the samples produced through the selective hydrothermal synthesis method revealed a very developed surface consisting of flake-shaped crystals with a thickness in the range of 15–20 nm and an average length of 150 nm in a predominantly vertical orientation (Figure 2a). The measurements of the surface profile using AFM allowed the average height of nanostructures to be determined within 100 nm (Figure 2a–c) for the nanostructured surface. The surface area is an essential factor for the efficient operation of electrolyte-gated FET devices due to the capacitance of the electric double layer, and thus, the application of nanostructures can significantly increase this parameter providing a better performance of the device at the same operating voltages. However, such a developed surface has pronounced hydrophobic properties, which greatly complicates the use of water-based analytes. The hydrophilicity and hydrophobicity of a surface are quantified by the contact angle θ. This angle is measured between the surface and the water inside the droplet. If θ < 90°, then the surface is hydrophilic. In this case, the surface tension at the interface of a solid body with water is less than at the interface of a solid body with air. The lower the contact angle, the more hydrophilic the surface. Water spreads on extremely hydrophilic surfaces. If θ > 90°, the surface is hydrophobic, and water will collect on the surface as droplets [35]. Hydrophobicity can also be considered as a small degree of hydrophilicity, since all substances have it to a greater or lesser extent [36].

Depending on the synthesis method, the surface of copper oxide can have either hydrophilic [37,38] or hydrophobic [39] properties, which can significantly affect the results of the experiment. The measurements were therefore carried out no earlier than 90 s after the introduction of 5 μL of water into the working area of the device for preliminary wetting of the surface, which ensured the leveling of undesirable effects caused by morphology.

**Figure 3:**
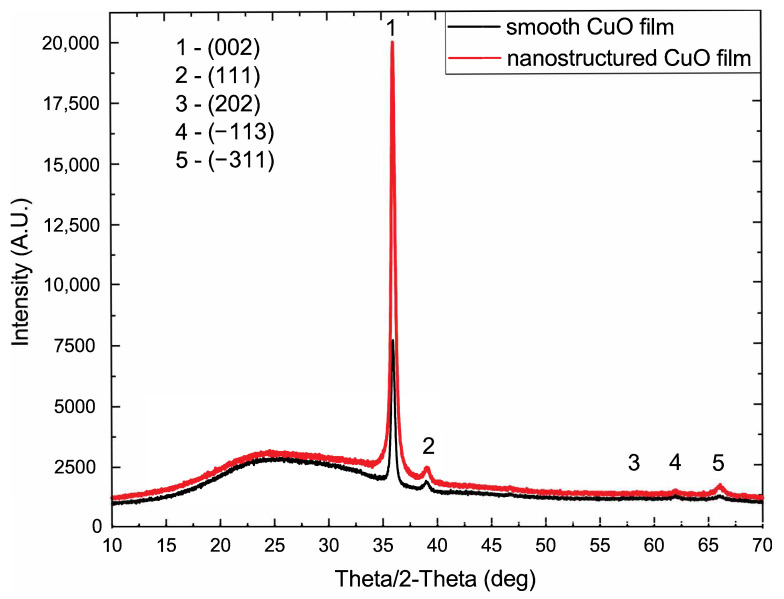
An XRD pattern of CuO nanostructured surface on polyamide substrate.

An XRD analysis of the smooth and nanostructured samples revealed a tenorite (CuO) phase of good crystallinity with a predominant orientation of (002) (Figure 3). A noticable amorphous background in the range of 15${}^{\circ }$ to 35${}^{\circ }$ theta/2-theta was due to the polymer substrate. Phase analysis was carried out using the “PDF-4” structural database.

### 3.3. Electrochemical Measurements

**Figure 4:**
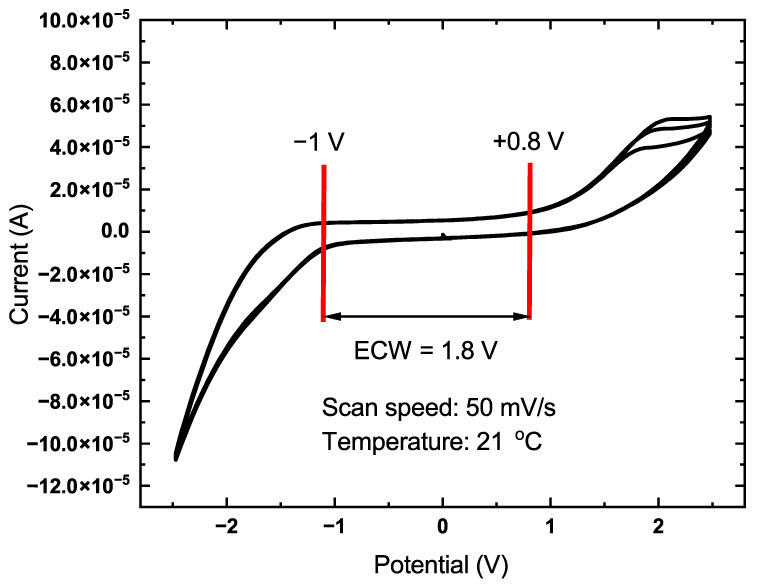
Cyclic voltammogram of the Au/DI-water/CuO system.

The determination of electrochemical properties, such as the size of the electrochemical stability window (ECW), is necessary for determining the maximum allowable voltage values applied to the electrodes of a TFT. Figure 4 presents the results of cyclic voltammetry of the Au/CuO electrodes with DI water used as an electrolyte for the nanostructured and smooth CuO samples. The electrochemical window does not have strictly defined boundaries and is determined by the region of the voltammogram with the lowest slope of current values. The scanning speed was chosen within 50 mV/s to minimize the effect of the EDL capacitance on the measurement results. The window size for this device was in the range from −1 to +0.8 V. Operation voltages beyond the ECW values would cause the rapid electrocorosion of the semiconductor material and water splitting that will adversely affect experimental results.

### 3.4. Electric Characterization

**Figure 5:**
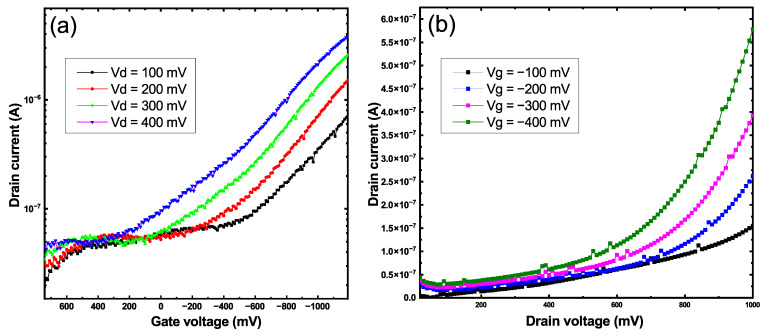
Transfer (**a**) and output (**b**) characteristics of the smooth CuO thin film transistor.

The transfer characteristics of the TFT with a 40 nm smooth thin-film channel are shown in Figure 5a. The “ON/OFF” ratio of the device reached 10${}^{2}$ at 0.4 V at the source-drain electrodes and −1 V at the gate acting as a p-type material. A shift of the threshold voltage to the positive side was observed when the voltage at the drain-source electrodes was increased; this is a common effect in TFTs caused by the accumulation of charge carriers on the grain boundaries. The output curves in Figure 5b only show the linear part of the characteristics over a certain voltage range. This effect could be caused by the low transport properties of the material as well as the excess thickness of the CuO film.

**Figure 6:**
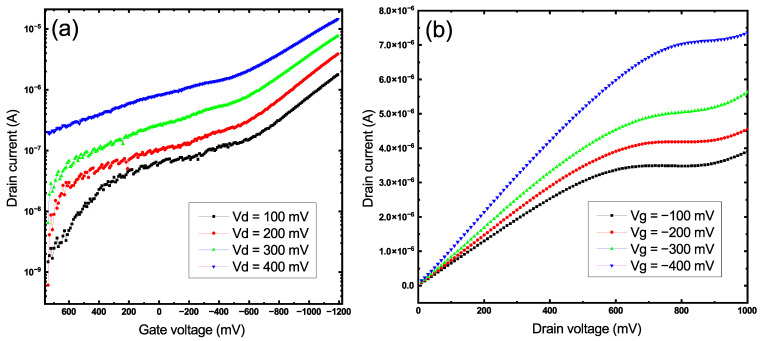
Transfer (**a**) and output (**b**) characteristics of the nanostructured CuO thin film transistor.

The transfer characteristics presented in Figure 6a were determined over a range of voltages from 0.4 V at the source-drain electrodes to −1 to +0.8 V at the gate according to the values of the electrochemical window for the Au/Cu system (Figure 4). An increase in the conductivity of the transistor channel in the region of negative values of the gate voltage was observed, which is typical for p-type semiconductors. During the measurements, a very long reaction time to a change in the applied voltage was revealed. Such an effect was most likely due to a large number of defects that acted as charge traps in the CuO structure [40]. To level the influence of this effect, the time interval between two measurement points was increased to 500 ms, and thus the duration of the entire measurement was 120 s. For the transistor with a nanostructured channel, the "ON/OFF" ratio was 10${}^{3}$ but starting from 350 millivolts at the source-drain, the channel experienced strong saturation, which led to a decrease in the field effect and a more linear behavior of the device (Figure 6a). Distinct linear and saturation regions starting around 600 mV at the source-drain electrodes were observed on the output curves of the transistor represented in Figure 6b unlike the smooth film FET device (Figure 5b), thus indicating a much better performance in terms of field effect.

### 3.5. Interaction of Analyte with FET

The data of electrochemical measurements, in particular cyclometry, revealed the size of the electrochemical window of about 1.8 V, and its main part lay in the negative voltage range. This represents an advantage since CuO is a natural p-type semiconductor, and the conductivity of FETs based on this material was increased by supplying a control signal of negative polarity to the gate of the device. It should be understood that the EDL is a non-ideal capacitor, which corresponds to the equivalent circuit of the constant phase element (leaky capacitor). In addition, the aim of these measurements was to determine the operating voltage range at which the leakage current would be minimal and, as a result, the minimum electrochemical corrosion rate of the semiconductor material. This phenomenon is usually a parasitic effect, but in this work, it was used as a source of Cu${}^{2+}$ ions for complexation with glyphosate.

Early-stage experiments were conducted in subthreshold operation mode, which according to reference [41], provides maximum sensitivity, but the noise level was too high for proper interpretation of the results. In this regard, higher source-drain and gate voltages were chosen as a “setpoint” (0.2 V${}_{DS}$ and −0.5 V${}_{G}$) to maintain similar current levels at the beginning of the experiment for both types of devices and secure more stable operation and higher resolution. A higher voltage leads to a more rapid emission of Cu${}^{2+}$ ions in the bulk of the electrolyte, which causes more intense Cu-glyphosate complex formation and, as a result, a higher resolution.

**Figure 7:**
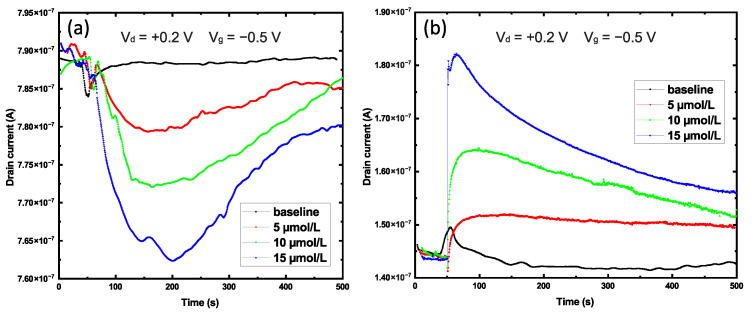
Time domain graphs representing the interaction of analytes of 3 different concentrations with smooth surface (**a**) and nanostructures (**b**).

An exposure time of 50 s after the voltage was applied allowed the device to reach the equilibrium mode of operation and to carry out a preliminary injection of Cu${}^{2+}$ ions into the volume of the analyte. After that, an analyte consisting of DI with dissolved glyphosate was added. The figure shows the results of the interaction of the transistor with three different concentrations of glyphosate in real time. Low concentrations of the analyte were chosen to minimize the change in the initial pH level of the analyte because glyphosate is an organic acid. After adding 5 μL of DI to the cuvette, there was a rapid increase in the value of the drain current caused by the perturbation in the EDL of the system, followed by a return to the initial state (i.e., the baseline; Figure 7b). In turn, when the glyphosate-containing analyte was added with a sharp increase in the current was observed, followed by its slow decline. The device exhibited a linear response to the three different concentrations. At maximum concentrations, the change in channel conductivity reached 19.42%. For LOD and sensitivity calculations, the extreme points for both graphs were taken. For a transistor with a channel in a smooth film, this is the 200th second from the beginning of the experiment, and for a nanostructured channel, it is the 70th second. Thus, for a FET based on a smooth CuO film LOD = 3.492 μM and sensitivity = 1.72 nA/μM. For FET with nanostructured channel LOD = 3.732 μM and sensitivity = 3.00 nA/μM. A comparative analysis of glyphosate sensors of similar types is summarized in Table 1.

An opposite behavior was observed for the samples with a smooth CuO film. After 150 s following the addition of a portion of the analyte containing any of the three concentrations of glyphosate, there was a decrease in the conductivity of the transistor channel, followed by a slow recovery until the end of the measurement (Figure 7a). The relative change in the conductivity of the channel reached 3.3%, i.e., it was 5.8 times lower than that of the nanostructured FET. Although holes are the predominant type of charge carriers for smooth and nanostructured CuO layers, the surface morphology of the layers can have a significant effect not only on the contact angle but also on the charge state of the surface, which directly affects the processes occurring in the EDL [47]. Reference [48] represents an example of a change in the increase in the value of the isoelectric point for TiO${}_{2}$ from 6.28 for a smooth film up to 3.47 for a nanostructured surface, explaining this effect of self-overlapping of EDLs as being caused by surface roughness. Apparently, this effect also takes place for surfaces based on CuO material.

**Figure 8:**
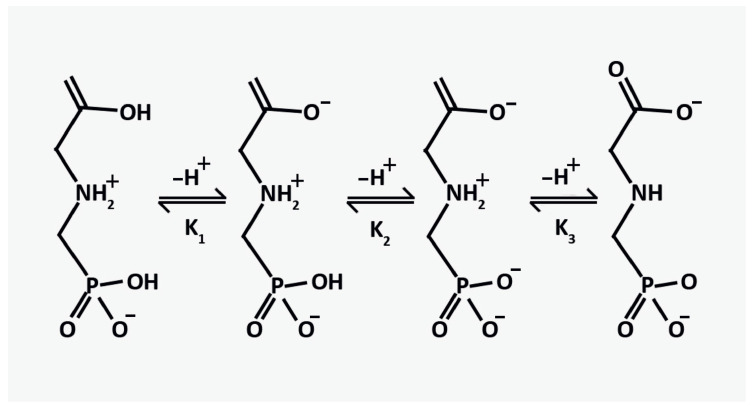
Scheme of dissociation of glyphosate in water.

The dissociation constants of N-phosphonomethylglycine or glyphosate take the following values: pKa1 0.8 (1st phosphonic), pKa2 2.3 (carboxylate), pKa3 6.0 (2nd phosphonic), and pKa4 11.0 (amine). It results in deprotonation of the acidic group and association with the most basic group, forming a dipolar molecule called a zwitterion [49]. It exists in different ionic forms depending on the pH of the environment, and its dissociation proceeds according to the scheme presented in Figure 8 [49].

Glyphosate molecules in solution are zwitterions, molecules with a dipole moment, which increases the total electrical capacitance of the system due to a higher dielectric permeability and, in turn, leads to an increase in the current within the transistor channel. The slow decrease in the drain current is caused by a change in the capacitance of the system due to the process of Cu-glyphosate complexation and its deposition on the CuO surface. Another important aspect is the dynamics of the pH level during the measurement process. However, direct measurements of pH are challenging due to the small volumes of the analyte in the working area of the device, but the initial pH of the 15 μmol/L analyte was equal to 6.05 due to the acidic properties of glyphosate.

In references [50,51], the authors studied Cu${}^{2+}$ and glyphosate herbicide complexes in an aqueous solution by means of pH measurements (over a temperature range of 5–45 °C), calorimetry, and visible spectrophotometry. Potentiometric data at these temperatures over a pH range of 2.5 to 10.5 explained the formation of the following forms: CuLH${}^{0}$, CuL${}^{-}$, CuLH${}^{2-}{\;}_{-1}$, CuL${}^{4-}{\;}_{+2}$, Cu${}_{2}$L${}^{+}$. As the pH level decreases, the negative net charge of the zwitterion decreases. Therefore, at the pH level of the solution equal to 4.5, the net charge of the glyphosate molecule has a value of 1- due to partial protonation, which inhibits the Cu–Glyp complex formation. At the same time, an acidic medium causes dissolution of the CuO surface, thus stimulating the injection of Cu${}^{2+}$ ions into the electrolyte. It may seem that higher pH levels that do not exceed the isoelectric point (9.5 for CuO) would provide more active glyphosate–copper complex formation due to Cu${}^{2+}$ ions and a positively charged CuO surface; however, at higher pH values, the CuO surface will be passivated with -OH groups inhibiting the injection of Cu${}^{2+}$ ions and Cu–Glyp interaction with the CuO surface. According to reference [52], the best option for studying Cu–Glyp complex formation is to use a medium such as PBS (phosphate buffer saline) with pH = 7.4.

The presence of Cu${}^{2+}$ in solution enhanced the adsorption of glyphosate for several reasons: glyphosate coordinates with Cu${}^{2+}$ quite strongly, and the resulting Cu–glyphosate complexes have a higher capacity for adsorption than free glyphosate; the adsorption of glyphosate can occur at sites where Cu${}^{2+}$ ions have previously been adsorbed, acting as a bridge between the surface and glyphosate [51]; pH decreases in the presence of Cu${}^{2+}$ ions and glyphosate adsorption increases, so a decrease in pH leads to the formation of glyphosate compounds with a lower negative charge, which are more easily adsorbed on oppositely charged areas of the surface [53]. These explain the slow recovery for both nanostructured and smooth surfaces observed in Figure 7.

## 4. Conclusions

The selective hydrothermal synthesis method was shown to be effective in terms of nanostructuring certain surface regions in order to increase the surface area and reactivity of electrolyte-gated sensor systems. The FET with a nanostructured CuO channel exhibited much better performance than the smooth thin film device due to its higher surface area. However, larger amounts of defects acting as charge traps in the body of nanostructured film delayed the response time of the device and caused the strong saturation of the transistor channel. In the case of the nanostructured channel, the addition of an analyte to the working area of the sensor led to a proportional increase in the conductivity of the channel, while the opposite effect was observed for samples with a smooth surface. In general, the FET sensor with a nanostructured CuO channel was 1.75 times more efficient than the devices with a smooth FET channel. The inversion effect of the analyte signal on the nanostructured surface was noted. This feature should be taken into account when designing sensors of this type. Another possible improvement of such sensor systems is downscaling the channel dimensions. However, in the case of a nanostructured surface, this will also lead to an increase in hydrophobic effects, which will adversely affect the signal-to-noise ratio of the device.

## Figures and Tables

**Table 1:** A summary of the properties of the various types of glyphosate sensors.

Type of Sensor	Active Surface	LOD (μM)	Reference
Electrochemical	Cu	0.003	[42]
Fluorescent	CuO/MWCNTs	4	[43]
Fluorescent	AChE	2.0	[44]
Colorimetric	AuNPs-Cys	0.15	[20]
Colorimetric	TMB	1.0	[45]
WG-OFET	P3CPT	0.13	[46]
WG-FET	CuO (film)	3.73	This study
WG-FET	CuO (nanostructures)	3.49	This study

## Data Availability

Not applicable.
